# Learning from wild *Vigna*: how should we develop a salt-tolerant crop?

**DOI:** 10.1270/jsbbs.25012

**Published:** 2025-08-09

**Authors:** Ken Naito, Fanmiao Wang

**Affiliations:** 1 Research Center of Genetic Resources, National Agriculture and Food Research Organization, 2-1-2 Kannondai, Tsukuba, Ibaraki 305-8602, Japan

**Keywords:** *Vigna*, wild crop relatives, salt tolerance, genetic control mechanisms

## Abstract

Salt tolerance has been an important issue in agriculture. Many genes involved in salt stress have been identified, but this knowledge has not led to development of a salt-tolerant crop that are practically useful. Despite hundreds of transgenic plants have been tested, there are few examples that demonstrated yield performance that are practically applicable to salt-affected fields. It is therefore important to figure out which genes should be targeted for artificial manipulation. However, given there are >500 of genes involved, it is almost impossible to test all the possible combinations of genes and expression profiles even in model plants. In contrast, wild plants inhabiting coastal environments have acquired salt tolerance, often by enhancing the mechanisms that are also conserved in model plants. Elucidating the mechanisms and underlying genes in such wild plants should provide a clear guidance to the combinations of appropriate genes. The genus *Vigna* represents such wild plants because of its great diversity. Recent studies have revealed that multiple *Vigna* species have independently evolved salt tolerance in various ways. Some of the species are studied in detail, highlighting the significance of combining and pyramiding multiple mechanisms for improving salt tolerance of a plant.

## Introduction

Stress tolerance is a key for feeding the ever-growing population. Abiotic stress could sometimes decline crop production by ~80% (Nehra *et al.* 2024), whereas biotic stress causes 20–40% yield loss every year (Savary *et al.* 2012). Moreover, the global climate change has been imposing more stress on plants. Besides heat and drought, it accelerates soil salinization by increasing evaporation and rising sea-level. It also brings more biotic stress by allowing expansion of new pests and diseases to regions of higher-latitude. Given the climate change does not seem to slow down, development of stress-tolerant varieties is urgently needed.

To challenge the issue, we have to harness the genetic potential of crop wild relatives and undomesticated wild species (McCouch *et al.* 2013). As wild plants have been repeatedly exposed to environmental extremes, they have evolved mechanisms to survive or even grow under such harsh conditions. However, their stress tolerance and adaptive capacity has largely remained unexplored. Now we have to open the door of genebank and explore the untapped genetic resources.

Being a reservoir of diversity, the genus *Vigna*, a member of Phaseoloid in the family Fabaceae, represents such genetic resources. It consists of more than 88 taxa, many of which inhabit harsh environments including marine beaches, poor sandy soils, or regularly submerged lands (Maxted *et al.* 2004, Tomooka *et al.* 2014) (see Table 1). With increasing demand for stress tolerance as described above, *Vigna* genetic resources have never been more important than they are today. Harnessing the genetic potential of wild *Vigna* could be a key to the development of stress-tolerant crops for feeding the future (Tomooka *et al.* 2011, van Zonneveld *et al.* 2020).

One simple strategy is to utilize the genetic resources as materials for conventional breeding. As the genus *Vigna* comprises multiple grain legumes including cowpea (*Vigna unguiculata* L. Walp), mungbean (*Vigna radiata* (L.) R. Wilczek) (*Vigna mungo* (L.) Hepper), adzuki bean (*Vigna angularis* (Willd.) Ohwi & H. Ohashi) and many others (Table 2), the tolerance and resistance can be introduced to the crops by crossing with the wild relatives.

However, one domesticated species is able to make hybrids with only its close relatives (Tomooka *et al.* 2014, van Zonneveld *et al.* 2020). To transfer the stress tolerance of wild *Vigna* to a broader range of crops, we have to elucidate the genetic mechanisms of wild species to learn how to utilize the genes involved in the tolerance mechanism. Although *Vigna* species have acquired various kinds of stress tolerance, they do so by enhancing the mechanisms that are broadly conserved across plant taxa. For example, the tolerance of *V. umbellata* to soil acidity and high aluminum is due to abundant expression of *Sensitive to Proton Rhizotoxicity 1* (*STOP1*) (Fan *et al.* 2015) and *Multidrug and Toxin Efflux Family 1* (*MATE1*) (Liu *et al.* 2013), both of which are known to play important roles in tolerance to low pH and high aluminum in Arabidopsis (Iuchi *et al.* 2007, Liu *et al.* 2009). Our recent studies (Noda *et al.* 2025, Wang *et al.* 2025) also revealed that *V. marina* has achieved its extraordinary salt tolerance by enhancing the well-known system; sodium antiporter activity (Gupta and Huang 2014) and root apoplastic barrier (Chen *et al.* 2011). According to these discoveries, upregulating endogenous genes could significantly improve stress tolerance of any kinds of crops.

The above strategy may seem redundant, given that research has already identified hundreds of stress-related genes from model plants. However, for example, there are few cases demonstrating the practical performance of transgenic plants that have altered expression of the genes involved in salt tolerance (Kotula *et al.* 2020). Since Flowers (2004) claimed that a decade of transgenic efforts had not established the value in the salt-affected field, hundreds of transgenic plants have been tested for salt tolerance. Although many produced 20%–50% more biomass or seeds than WT under salt stress (100–250 mM NaCl), the performance of transgenics have actually declined by ~50% under salt stress compared to the non-saline condition. Typically, Pehlivan *et al.* (2016) demonstrated that a transgenic plant co-overexpressing *Salt Overly Sensitive 1* (*SOS1*) and *Na^+^/H^+^ Exchanger 1* (*NHX1*), encoding plasma membrane and vacuolar membrane sodium/proton antiporter, respectively, produced 300% more seeds than WT under a condition of 250 mM NaCl. However, compared to WT under the non-saline condition, the seed production declined by >90% in the transgenic plants under salt stress (Pehlivan *et al.* 2016). In addition, altering expression of a stress-responsive transcription factors often dramatically improve stress tolerance but causes negative impacts including growth retardation, as exemplified in the case of Dehydration Responsive Element Binding protein 1A (DREB1A) (Liu *et al.* 1998). These examples indicate that altering one or two genes hardly achieves salt tolerance that is required for practical use in salt-affected fields. To overcome these problems, we need a protocol on which set of genes should be selected and when and where they should be expressed. It is wild *Vigna* where we can find such a protocol by elucidating the mechanisms of stress tolerance.

Here we review the recent updates in the studies of salt tolerance in the wild *Vigna*. Though the issue of climate change has recently drawn more interest on drought and heat tolerance, salt tolerance is still important to challenge the issues of not only soil salinization but also of freshwater shortage. In some regions the amount of groundwater drawn for irrigation exceeds 20 times the amount that is recharged (Wada *et al.* 2010). One solution to save freshwater is saline agriculture, which utilizes brackish/saline water for crop cultivation (Rozema and Flowers 2008). To achieve this goal, salt-tolerant accessions have been selected from the *Vigna* genetic resources (Iseki *et al.* 2016, Yoshida *et al.* 2016). The selected accessions exhibit great diversity in the mechanisms of salt tolerance, revealing there are various options for a plant to acquire salt tolerance. The following studies investigated some of the mechanisms in detail, having identified the underlying genes. This review also discusses future strategies for developing salt-tolerant crops by integrating various mechanisms of salt tolerance identified in wild *Vigna*.

## Performance of the salt-tolerant *Vigna*s under salt stress

### Vigna nakashimae 

In 100 mM NaCl, *V. nakashimae* does not show any symptoms of salt damage for >4 weeks while *V. angularis* gets completely wilted within 2 weeks (Yoshida *et al.* 2016). It maintains chlorophyll fluorescence even in 200 mM NaCl (Iseki *et al.* 2016), but its photosynthesis significantly declines under 100 mM NaCl in greenhouse (Yoshida *et al.* 2016). However, it produced even more biomass in a salinized field, in which soybean cultivars did not grow at all, than in a de-salinized field (Yoshida *et al.* 2016). In nature, it lives in hilly grasslands facing the ocean.

### Vigna riukiuensis 

**Fig. 1.**
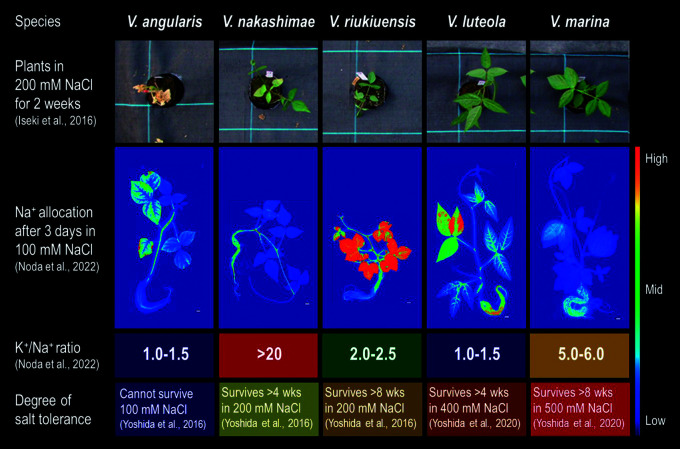
Overview of salt-tolerant *Vigna*s. The color scale indicates amounts of sodium allocation, K^+^/Na^+^ ratio and degree of salt tolerance.

*V. riukiuensis* often shows higher salt tolerance than *V. nakashimae*, which is its sister species, except it grows slower than many others (Iseki *et al.* 2016, Yoshida *et al.* 2016) (Fig. 1). It survives for more than 8 weeks in 200 mM NaCl and its photosynthesis does not greatly decline in 100 mM NaCl. It is also significantly resilient to a sudden initiation of intense salt stress (transfer to 200 mM NaCl without preculture with 50 mM NaCl) (Noda *et al.* 2023). It produced 5 times more biomass in the salinized field than in the de-salinized field (Yoshida *et al.* 2016). In nature, it lives in tropical hilly grasslands facing the ocean.

### Vigna luteola 

*V. luteola* is significantly more tolerant to salt stress than *V. nakashimae* or *V. riukiuensis*. One accession of *V. luteola* survives more than 4 weeks under 400 mM NaCl without a sign of salt damage (Yoshida *et al.* 2020). It also maintains transpiration and photosynthetic activity under 150 mM NaCl, which is almost comparable to the control condition (Yoshida *et al.* 2020). In nature, it usually lives in marine beaches but sometimes in riverbanks.

### Vigna marina 

*V. marina* is also a sister species of *V. luteola* but shows even better performance under salt stress. An accession of *V. marina* does not show salt damage even after 4 weeks of salt stress with 500 mM NaCl (Yoshida *et al.* 2020). It even increases transpiration and photosynthetic activity in response to 150 mM NaCl (Yoshida *et al.* 2020), and changes root morphology to maintain water uptake against osmotic pressure of 200 mM NaCl (Wang *et al.* 2024). In nature, it lives specifically in marine beaches.

## Ion allocations in the salt-tolerant *Vigna*s

As several species independently evolved to adapt themselves to saline environments, the mechanisms of salt tolerance are often different from each other (Iseki *et al.* 2016, Noda *et al.* 2022, Yoshida *et al.* 2016, 2020). The most outstanding difference is in Na^+^ allocation, which is well visualized by autoradiograph using radioactive Na (^22^Na) (Fig. 1). *V. nakashimae* allocates Na^+^ to the root or the lower stem but not to the leaf or upper stem including the shoot apical meristem (SAM), whereas *V. riukiuensis* does to the leaf and SAM, which resembles the pattern of salt-sensitive plants. *V. luteola* allocates Na^+^ to the root and particularly the topmost fully-expanded leaf, whereas *V. marina* keeps Na^+^ allocation low even in the root.

Though salt stress inhibit uptake of essential ions including K^+^, Mg^2+^ and Ca^2+^ (Parihar *et al.* 2015), the salt-tolerant *Vigna*s are able to maintain the transport of such ions (Noda *et al.* 2022, Wang *et al.* 2025, Yoshida *et al.* 2020). In particular, *V. nakashimae* and *V. marina* accumulates higher amount of K^+^ in the shoot, especially in the leaves and upper shoot where Na^+^ allocation is strongly restricted (Noda *et al.* 2022, Wang *et al.* 2024, Yoshida *et al.* 2020). *V. marina* also shows superior ability in transporting Mg^2+^ and Ca^2+^ compared to other salt-tolerant species (Wang *et al.* 2025, Yoshida *et al.* 2021). These facts suggest that salt tolerance needs not only control or restrict Na^+^ allocation, but also maintain transport of essential ions.

## Mechanisms of tolerance and the underlying genes in the salt-tolerant *Vigna*s

### Vigna nakashimae 

**Fig. 2.**
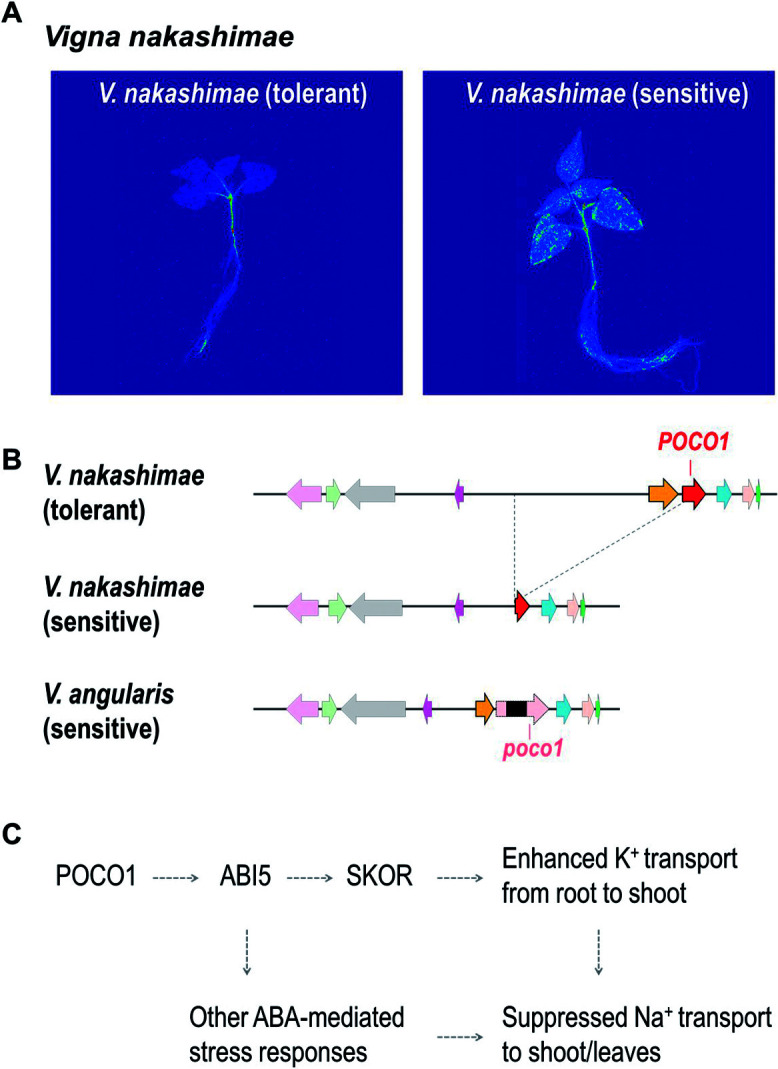
Overview of salt tolerance mechanism in *Vigna nakashimae*. A. Autoradiographs ^22^Na-treated plants of the salt-tolerant and salt-sensitive accessions of *V. nakashimae*. B. Schematic of a genomic region involved in salt tolerance. C. Presumed model of *V. nakashimae*’s response to salt stress.

A genetic study on salt tolerance of *V. nakashimae* has identified a single QTL with a large effect on Chr08 (Ito *et al.* 2024). This study utilized an intraspecific variation of salt tolerance in *V. nakashimae*, as the accessions from Ukushima Island are salt-tolerant whereas many others are not (Fig. 2A) (Ito *et al.* 2024, Ogiso-Tanaka *et al.* 2024). The following whole genome sequencing and transcriptome analysis revealed that the sensitive accessions have a ~50 kbp deletion that disturbs the promoter and the 5ʹ end of *PRECOCIOUS 1* (*POCO1*) gene (Fig. 2A). Further comparative analysis also revealed that *V. angularis*, which is also salt-sensitive, lacks this gene by insertions of transposable elements (Fig. 2B). *POCO1* is a positive regulator of *ABA Insensitve 5* (*ABI5*) (Emami and Kempken 2019) and the *poco1* mutant have lower expression of ABA-responsive genes in Arabidopsis (Emami *et al.* 2020). Thus, it could also positively regulate salt tolerance (Fig. 2C).

The study also implies the role of *POCO1* in K^+^ transport. In the tolerant accession, *Stelar K^+^ Outward Rectifier* (*SKOR*) is highly expressed together with *POCO1* (Ito *et al.* 2024). *SKOR* is positively regulated by ABA and is involved in K^+^ transport from the root to the shoot (Demidchik 2014). Thus, ABA-signaling could be upregulated in the tolerant accession including K^+^ transport by *SKOR* (Fig. 2C), which may explain the high ability of maintaining K^+^/Na^+^ ratio in the tolerant accessions of *V. nakashimae* (Iseki *et al.* 2016, Noda *et al.* 2022) (Fig. 1).

### Vigna riukiuensis 

**Fig. 3.**
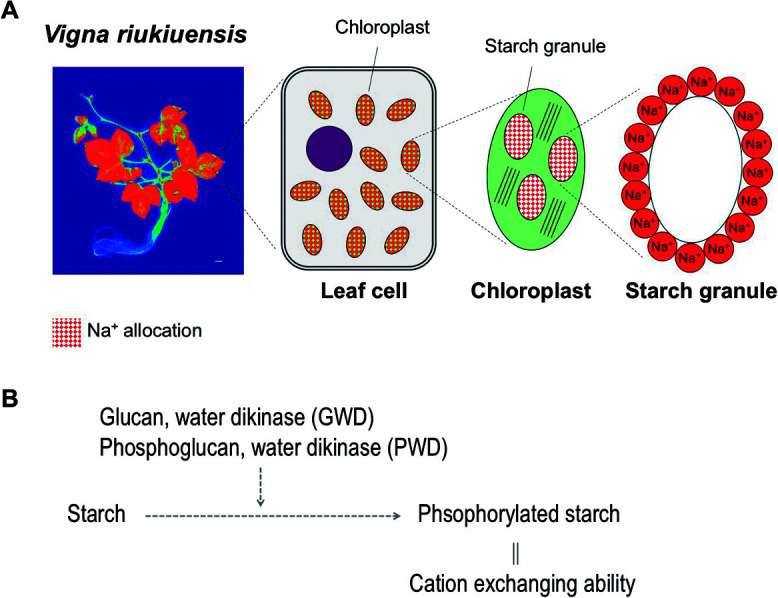
Overview of salt tolerance mechanism in *Vigna riukiuensis*. A. Illustration of starch-dependent Na^+^ isolation. B. Presumed model of producing Na^+^-binding starch granules.

*V. riukiuensis* allocates high amounts of Na^+^ in the leaf because it accumulates lots of starch granules with Na^+^-binding activity in the chloroplast (Fig. 3A) (Noda *et al.* 2023). As the starch granules trap and lower the free Na^+^ in the cytosol, the essential enzymatic activities are protected from the ion toxicity. Similarly, common reed (*Phragmites australis*) forms Na^+^-trapping starch granules in the stem to suppress the Na^+^ allocation to leaves (Kanai *et al.* 2007). Though starch is not usually considered to bind ions, plant starch has negative charges due to phosphate groups added to hydroxyl groups in the alpha-glucan chains. Potato starch, which has a higher rate of phosphorylation than cereal starch, has higher contents of cations including Na^+^ and K^+^ (Zaidul *et al.* 2007). Given starch phosphorylation is usually mediated by Glucan, Water Dikinase (GWD) and Phosphoglucan, Water Dikinase (PWD) (reviewed by You *et al.* 2020), the genes encoding these proteins may be upregulated in *V. riukiuensis*. Thus, although not yet thoroughly investigated, the starch granules in *V. riukiuensis* are capable of trapping Na^+^ probably due to higher rate of phosphorylation.

### *Vigna marina* and *Vigna luteola*

*V. marina* and *V. luteola* are both distributed in marine beaches and share some features of salt tolerance as below.

1. High Na^+^ excretion from roots by SOS1 (Noda *et al.* 2025)

2. Diurnal regulation of the SOS pathway by diurnally-regulated SOS2 (Noda *et al.* 2025)

3. Low Na^+^ uptake by endodermal apoplastic barrier (Wang *et al.* 2025)

The differences currently known between the two species are in 1 and 3; *V. marina* is able to excrete more Na^+^ (Noda *et al.* 2025) and develop a thicker apoplastic barrier (Wang *et al.* 2025).

Compared to salt-sensitive species including *V. angularis*, *V. marina* excretes significantly more Na^+^ from the root (Noda *et al.* 2025). *V. luteola* also excretes more Na^+^ than *V. angularis* but not so much as *V. marina* (Noda *et al.* 2025). The transcriptomic studies indicate a strong correlation between Na^+^ excretion and the expression of *SOS1*. The *SOS1* locus of *V. marina* is associated with salt tolerance in the F_2_ plants derived from *V. marina* and *V. luteola* and contains a TE insertion in the promoter. This insertion may have introduced *cis*-elements that could be responsible for the constitutive expression of *SOS1* (Noda *et al.* 2025).

Furthermore, *V. marina* and *V. luteola* both excrete Na^+^ only during the light period and not during the dark period, unlike *V. angularis* with no diurnal patterns (Noda *et al.* 2025). *SOS2* expression also follows a diurnal pattern in *V. marina* and *V. luteola*, being higher in the light and lower in the dark period. In contrast, *V. angularis* does not show diurnal patterns in *SOS2* expression. As *SOS2* encodes a CBL-Interacting Protein Kinase (CIPK) and positively regulates SOS1 activity through phosphorylation, its expression plays a pivotal role in the diurnal regulation of Na^+^ excretion. Both *SOS1* and *SOS2* are involved in the well-known system of Na^+^ homeostasis, the SOS pathway (reviewed by Ji *et al.* 2013). As described above, *SOS1* encodes one of the Na^+^/H^+^ antiporters that are located on a plasma membrane, extruding Na^+^ from the cytosol in exchange for H^+^. The antiporter activity relies on phosphorylation mediated by SOS2, which also needs interaction with another protein SOS3. *SOS3* encodes a Calcineurin B Like protein, which serves as a Ca^2+^ sensor and triggers various kinds of stress signaling.

**Fig. 4.**
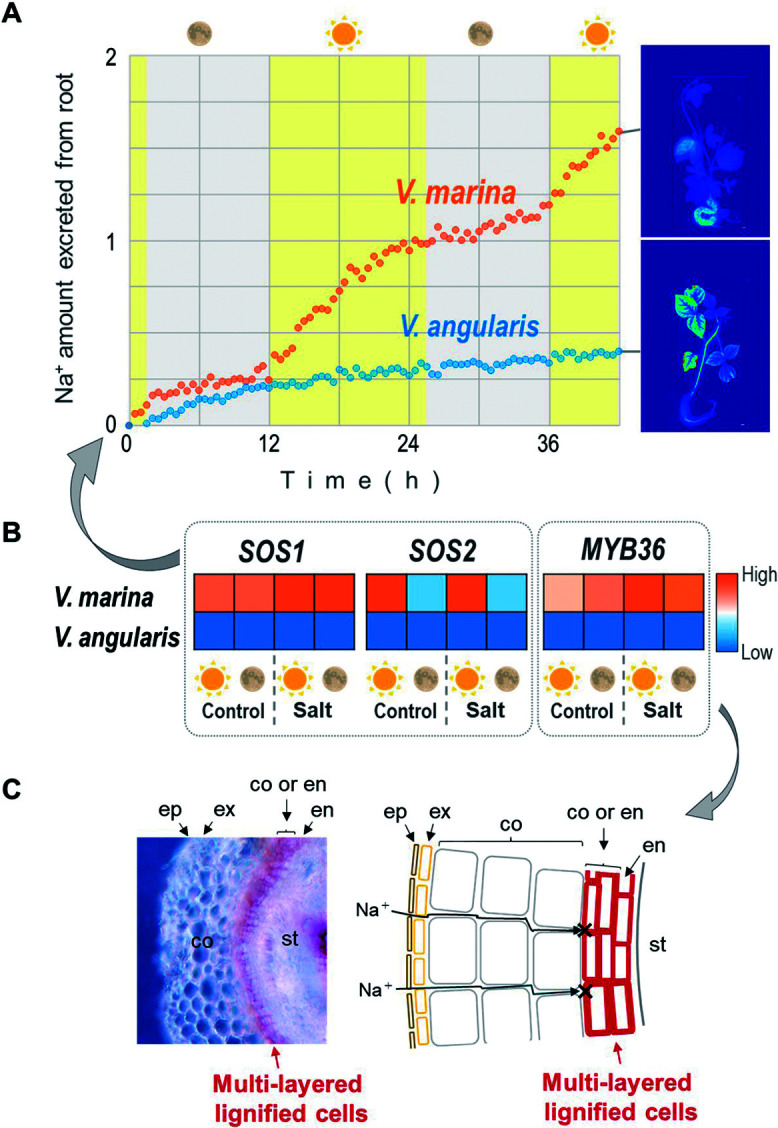
Overview of salt tolerance mechanism in *Vigna marina*. A. Na^+^ excretion across time in *V. marina* and *V. angularis*. Yellow and gray indicate daytime and nighttime, respectively. B. Heatmap of *SOS1*, *SOS2*, and *MYB36* expression. The icons of the sun and the moon indicate daytime and nighttime, respectively. The color scale indicates the relative expression level. C. Stereoscopic photograph and illustration of *V. marina*’s root section. ep, ex, co, en and st indicate epidermis, exodermis, cortex, endodermis and stele, respectively.

Along with the remarkable capacity for Na^+^ excretion, the apoplastic barrier significantly suppresses transpiration-dependent Na^+^ uptake in *V. marina* and *V. luteola* (Fig. 4C) (Wang *et al.* 2025). Apoplastic barrier is often called Casparian strip, which is typically a band-like structure in the center of root endodermis (Barbosa *et al.* 2019). It is mainly composed of lignin and suberin, both of which are crucial for restricting apoplastic flow across endodermis (from cortex to vascular bundle). This forces water and solutes to pass the endodermis by symplastic transport, filtering out unwanted substances. Recent studies have highlighted the role of lignin, not suberin, in limiting transpiration-dependent Na^+^ uptake in many plants (Calvo-Polanco *et al.* 2021, Reyt *et al.* 2021, Wang *et al.* 2022). Similarly, *V. marina* and *V. luteola* also form apoplastic barrier in response to salt stress (Wang *et al.* 2025). However, while *V. luteola* forms a typical, single-layered Casparian strip in endodermis, *V. marina* accumulates lignin in the apoplastic space around endodermis and forms a multi-layered barrier, which is more than a strip (Fig. 4C) (Wang *et al.* 2025). The multi-layered barrier of *V. marina* strictly blocks the apoplastic flow of Na^+^ even in transpiring conditions such as tropical marine beaches.

**Fig. 5.**
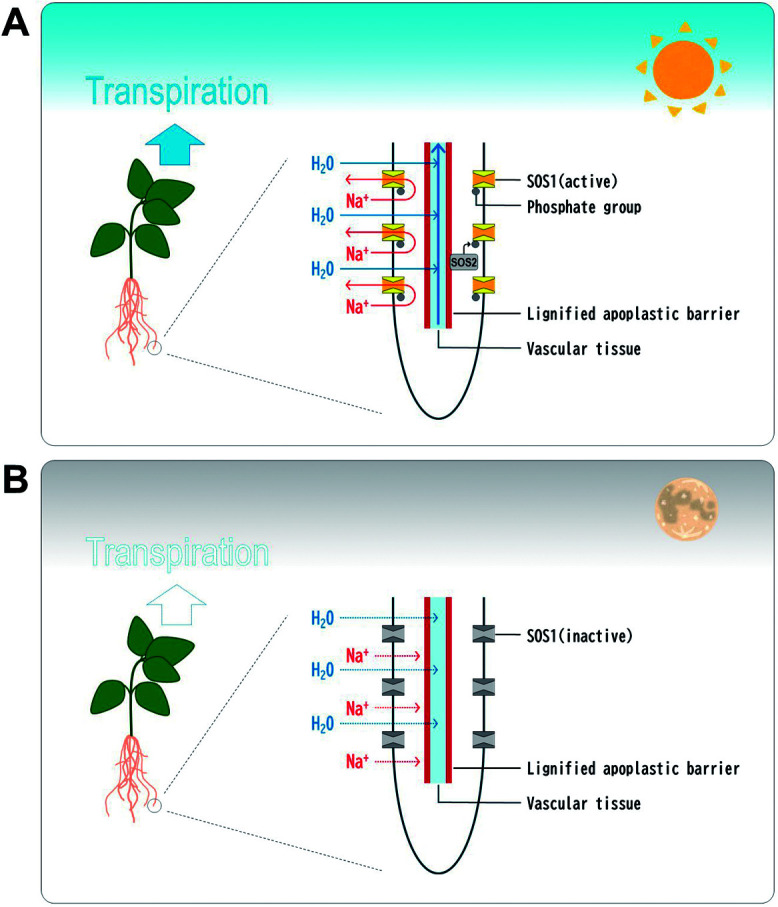
Presumed model of water and Na^+^ dynamics in *V. marina*. A. Daytime: Opened stomata trigger transpiration-dependent water uptake, drawing water and Na^+^ into the root. But the apoplastic barrier blocks Na^+^ ions and let only water be transported to the leaf through xylem. In addition, the upregulated SOS2 turns SOS1 on, triggering active excretion of Na^+^ out of the root. B. Nighttime: Closed stomata stops transpiration-dependent water uptake. Though Na^+^ may passively flow into the root, the apoplastic barrier still blocks Na^+^. Given transpiration is stopped, the downregulated SOS2 turns SOS1 off, saving the energy cost of SOS pathway.

With all these features integrated, the mechanism of salt tolerance in *V. marina* is as follows (Fig. 5): During daytime, Na^+^ enters root by transpiration-dependent water flow but is blocked by the thick apoplastic barrier at endodermis. Meanwhile, the upregulated SOS2 proteins turn on SOS1 proteins, which are constitutively expressed, and trigger active pumping the filtered Na^+^ out of the root. During nighttime, though transpiration is stopped, Na^+^ may passively enter the root along with the gradient of Na^+^ concentration but cannot reach the vascular bundles because of the apoplastic barrier. This allows the plant to turn off SOS1 by downregulating SOS2 and avoid wasting excess energy.

## Perspectives

### Importance of model plants as reference

Though we have focused on the studies of wild plants in this article, the model plants have been and will be an important reference. Because studies on model plants have elucidated various aspects of stress response, we have been able to successfully identify the mechanisms in wild *Vigna*s (Figs. 1–5). Given the whole picture of stress response is not yet known in model plants, we have to keep studying them to make the reference more comprehensive. The more comprehensive reference will further facilitate pointing out which part of the picture is important in the stress tolerance that has evolved in wild plants.

### Combining multiple mechanisms

The studies on *V. marina* indicated the importance of integrating multiple mechanisms. To achieve the extraordinary salt tolerance, *V. marina* has enhanced its ability of the root to block and excrete Na^+^. While the *sos1* mutant is hypersensitive to salt stress (Wu *et al.* 1996), the plants overexpressing *SOS1* are not necessarily more tolerant than WT (Kotula *et al.* 2020). This could be because *SOS1* cannot solely manage the apoplastic flow of Na^+^, as Wang *et al.* (2025) showed that disturbing endodermal barrier, but not *SOS1* expression, significantly increased shoot Na^+^ allocation and salt damage in *V. marina*. Thus, the SOS1 activity and apoplastic barrier have synergistic effects. This exemplifies which set of genes should be targeted to improve salt tolerance of a plant.

**Fig. 6.**
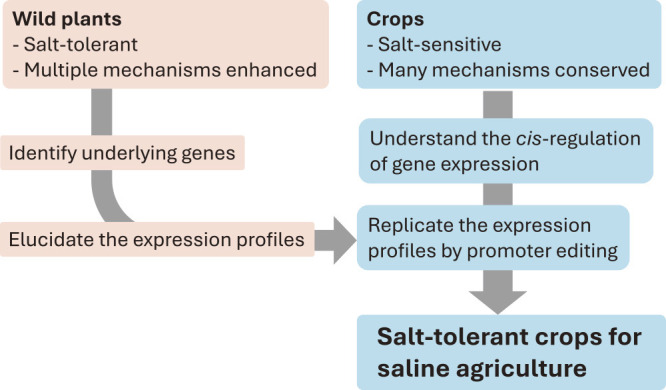
Model of future crop design for salt-tolerant crops.

Moreover, *V. nakashimae* and *V. riukiuensis* have also evolved their own mechanisms of salt tolerance. While *V. nakashimae* has enhanced the ability of K^+^ transport under salt stress (Fig. 2) (Ito *et al.* 2024), *V. riukiuensis* has invented a specific starch to isolate Na^+^ in leaves (Fig. 3) (Noda *et al.* 2023). We expect that these mechanisms have additional effects if combined with those elucidated in *V. marina*. Thus, to develop a stress-tolerant crop, it would be a viable strategy to combine and pyramid the mechanisms that are “enhanced” in wild plants for adaptation to harsh environments (Fig. 6).

### Importance of *cis*-element evolution

Since Wray *et al.* (2003) declared transcriptional regulation by *cis*-elements has to be the mainstream of molecular evolutionary study, it has been a hot topic especially in the field of evo-devo (Carroll 2008, Kim and Wysocka 2023, Long *et al.* 2016, Signor and Nuzhdin 2018, Vierstra *et al.* 2014, Wittkopp and Kalay 2012, Wray 2007). Given transcriptional factors are often involved in regulating hundreds of genes, mutations altering binding specificity could be catastrophic. In contrast, changes in *cis*-elements of downstream genes do not affect the protein function but affect their expression profile in time, space, and quantity, constituting a major component of genetic basis for phenotypic evolution (Wray *et al.* 2003). Though the studies had been mainly of animals and insects, plant scientists now recognize the importance of *cis*-elements in plant evolution (Marand *et al.* 2023, Schmitz *et al.* 2022, Yocca and Edger 2022).

Likewise, *cis*-element evolution should also be important in evolution of stress tolerance. As described in other sections in this article, wild *Vigna* species have evolved salt tolerance by changing gene expression profiles rather than altering protein sequences or acquiring new genes (Noda *et al.* 2025, Wang *et al.* 2025; https://doi.org/10.1101/2022.03.28.486085). These findings underscore the significance of *cis*-element evolution, as *V. marina* has acquired elevated *SOS1* expression due to a TE insertion in the promoter (Fig. 4B) (Noda *et al.* 2025). *V. marina* and *V. luteola* could also have evolved the diurnal regulation of *SOS2* by acquiring the specific promoter sequences, which are not present in other species, before the two species have diverged from the common ancestor (Fig. 4B) (Noda *et al.* 2025). Given the SOS pathway is conserved across a broad range of seed plants (Ismail and Horie 2017), the alteration of the *SOS1*/*SOS2* expression profiles should have been the key events in the evolution of their salt tolerance. In addition, *V. marina* has acquired the thickened apoplastic barrier by increasing the basal expression levels or salt-responsiveness of Casparian strip-related genes (Fig. 4B, 4C) (Wang *et al.* 2025). The salt tolerance of *V. nakashimae* and *V. riukiuensis* could also be attributed to the modified expression profiles of existing genes (Ito *et al.* 2024, Noda *et al.* 2023). Thus, evolution of stress tolerance does not necessarily need evolution of protein-coding sequences.

Given the importance of *cis*-element evolution, editing promoter sequences of endogenous genes could potentially enhance salt tolerance of a plant (Fig. 6). Many plant genomes, including those of crops, retain the important set of genes involved in Na^+^ transport, Casparian strip formation and many other mechanisms required for salt tolerance. Thus, it may not be necessary to introduce the genes from wild species into crops through transformation. Instead, editing the promoter sequences of endogenous genes would be a more practical approach to improve stress tolerance, as prime editing is now efficiently performed in various crops (Molla *et al.* 2021).

One limitation to be overcome is that the *cis*-regulation of plant genes are largely left unknown. Constitutive overexpression is often not an optimal approach, as overexpression of *MYB36*, which initiates Casparian strip formation, leads to ectopic lignification and notable growth retardation (Fernández-Marcos *et al.* 2017). In another example, the constitutive overexpression of *High-affinity K^+^ Transporter 1* (*HKT1*), which is involved in unloading Na^+^ from xylem sap, significantly reduces salt tolerance (Huang *et al.* 2020). Because HKT1 imports Na^+^ into cytosol, its overexpression would lead to Na^+^ accumulation throughout the plant body. Thus, we need to know the grammar of gene regulation in crop species as well as the cellular-level expression profile of the stress-related genes in wild species (Fig. 6). The accumulating evidence of single-cell transcriptome and epigenome studies will help develop technologies to reproduce the expression profile of wild genes in crops.

## Author Contribution Statement

KN and WF wrote the manuscript.

## Figures and Tables

**Table 1.** List of *Vigna* species reported to have stress tolerance

Species	Type of tolerance	Reference
*V. aconitifolia*	Drought	van Zonneveld *et al.* 2020, Iseki *et al.* 2018, Godbole *et al.* 1984
	Heat	van Zonneveld *et al.* 2020, Sharma *et al.* 2014
*V. aridicola*	Drought	van Zonneveld *et al.* 2020, Iseki *et al.* 2018
	Heat	van Zonneveld *et al.* 2020
*V. exilis*	Drought	Iseki *et al.* 2018, Takahashi *et al.* 2015
	Heat	van Zonneveld *et al.* 2020
	High pH	Takahashi *et al.* 2015
*V. hainiana*	Heat	van Zonneveld *et al.* 2020
*V. indica*	High pH	https://doi.org/10.1101/2022.03.28.486085
*V. lobatifolia*	Drought	Iseki *et al.* 2018
	Heat	van Zonneveld *et al.* 2020
*V. luteola*	Salt	Iseki *et al.* 2016, Yoshida *et al.* 2020, 2021, Noda *et al.* 2022, 2025, Wang *et al.* 2024, 2025
	Flooding	Bruce and Burt 1984
*V. marina*	Salt	Iseki *et al.* 2016, Yoshida *et al.* 2020, 2021, Noda *et al.* 2022, 2025, Wang *et al.* 2024, 2025
	High pH	Takahashi *et al.* 2014
*V. minima*	Low pH	Tomooka *et al.* 2002
*V. mungo*	Flooding	https://doi.org/10.1101/2022.03.28.486085
*V. nakashimae*	Salt	Iseki *et al.* 2016, Noda *et al.* 2022, Yoshida *et al.* 2016, Ito *et al.* 2024, Ogiso-Tanaka *et al.* 2024
*V. riadiata* var. *sublobata*	Heat	van Zonneveld *et al.* 2020
*V. riukiuensis*	Salt	Iseki *et al.* 2016, Noda *et al.* 2022, 2023, Yoshida *et al.* 2016, Ogiso-Tanaka *et al.* 2024
*V. stipulacea*	Flooding	Tomooka *et al.* 2002
*V. trilobata*	Drought	van Zonneveld *et al.* 2020, Iseki *et al.* 2018
	Salt	Iseki *et al.* 2016
	Heat	van Zonneveld *et al.* 2020
*V. umbellata*	Low pH	Yang *et al.* 2006, 2011, Liu *et al.* 2013, Fan *et al.* 2015
	High pH	Takahashi *et al.* 2015
*V. unguiculata*	Heat	van Zonneveld *et al.* 2020
*V. vexillata*	Drought	Iseki *et al.* 2018
	Salt	Iseki *et al.* 2016, Yoshida *et al.* 2020, 2021, Dachapak *et al.* 2019, Laosatit *et al.* 2024
	Flooding	Miller and Williams 1981
	Low pH	Tomooka *et al.* 2014
*V. vexillata* var. *ovata*	Heat	van Zonneveld *et al.* 2020
	Salt	Iseki *et al.* 2016

**Table 2.** List of domesticated *Vigna*

Species	Common name	Uses
*Vigna aconitifolia* (Jacq.) Marechal	Moth bean	Pulse, green pods, forage, cover crops, green manure (Maxted *et al.* 2004)
*Vigna angularis* (Willd.) Ohwi & H. Ohashi	adzuki bean	Pulse, green pods (Tomooka *et al.* 2002)
*Vigna glabrescens* Maréchal, Mascherpa & Stainier	Creole bean	Pulse (Tomooka *et al.* 2002)
*Vigna mungo* (L.) Hepper	Black gram	Pulse, green pods, forage, green manure (Maxted *et al.* 2004), sprout (Tomooka *et al.* 2002)
*Vigna radiata* (L.) R. Wilczek	Mung bean	Pulse, sprout (Maxted *et al.* 2004)
*Vigna subterranean* (L.) Verdc.	Bambara groundnut	Pulse, green pods (Maxted *et al.* 2004)
*Vigna umbellata* (Thunb.) Ohwi & H. Ohashi	Rice bean	Pulse, green pods, forage, green manure (Tomooka *et al.* 2002)
*Vigna unguiculata* L. Walp	Cowpea	Pulse (Maxted *et al.* 2004)
*Vigna unguiculata* subsp. *sesquipedalis* (L.) Verdc.	Yardlong bean	Green pods (Tomooka *et al.* 2002)
*Vigna vexillata* (L.) A. Rich.	Zombi pea	Tuber (Karuniawan *et al.* 2006)
